# Fourier Domain Mode Locked Laser and Its Applications

**DOI:** 10.3390/s22093145

**Published:** 2022-04-20

**Authors:** Dongmei Huang, Yihuan Shi, Feng Li, P. K. A. Wai

**Affiliations:** 1 Photonics Research Institute, Department of Electrical Engineering, The Hong Kong Polytechnic University, Hong Kong, China; meihk.huang@polyu.edu.hk (D.H.); yihuan.shi@polyu.edu.hk (Y.S.); 2 Shenzhen Research Institute, The Hong Kong Polytechnic University, Shenzhen 518057, China; alexwai@hkbu.edu.hk; 3 Photonics Research Institute, Department of Electronic and Information Engineering, The Hong Kong Polytechnic University, Hong Kong, China; 4 Department of Physics, Hong Kong Baptist University, Hong Kong, China

**Keywords:** swept laser, Fourier domain mode-locking, instability, frequency/time discretization, laser and optics system

## Abstract

The sweep rate of conventional short-cavity lasers with an intracavity-swept filter is limited by the buildup time of laser signals from spontaneous emissions. The Fourier domain mode-locked (FDML) laser was proposed to overcome the limitations of buildup time by inserting a long fiber delay in the cavity to store the whole swept signal and has attracted much interest in both theoretical and experimental studies. In this review, the theoretical models to understand the dynamics of the FDML laser and the experimental techniques to realize high speed, wide sweep range, long coherence length, high output power and highly stable swept signals in FDML lasers will be discussed. We will then discuss the applications of FDML lasers in optical coherence tomography (OCT), fiber sensing, precision measurement, microwave generation and nonlinear microscopy.

## 1. Introduction

In 1960, the first laser was invented by Theodore Maiman at Hughes Research Institute [1], which ignited the interest in the study and applications of lasers. Many Nobel prizes in Physics were awarded to researchers for their contributions to the development of lasers. The lasers have the advantages of monochrome, coherence, directionality, and high brightness, which have been widely used in medicine, material processing, optical communication and sensing. Despite the versatility of single-wavelength lasers, some applications call for pulsed lasers or swept lasers. In the frequency domain, the swept laser covers a wide output spectrum while maintaining the narrow line-width characteristic of single-wavelength sources [2,3]. In the time domain, the output wavelength of the laser is correlated with time, i.e., the wavelength information can be obtained directly from the temporal waveform detected by a high-speed oscilloscope. The wavelength scanning characteristics of the swept laser find wide applications in optical fiber sensing [4,5,6,7,8], laser measurement [9,10], swept-source OCT [11,12,13,14,15,16,17,18,19,20,21,22,23,24,25,26,27,28,29] and LiDAR [30,31]. Sweep rate, sweep range and instantaneous linewidth are three key parameters of swept lasers. The sweep rate represents the rate of change of the wavelength with time, the sweep range determines the detected spectral range and the instantaneous linewidth represents the coherence length.

A straightforward realization of the wavelength-swept source is to insert a tunable optical bandpass filter into a conventional laser cavity [2]. The tuning speed of such filter-guided swept sources is fundamentally limited by the buildup time of the new laser signals from spontaneous emissions after the filter is tuned to a new wavelength. The typical sweep rate of such tunable lasers is ~1 kHz. To speed up the rebuilding time of the laser signal, one method is to reduce the length of the laser cavity [32,33]. The sweep rate of a short cavity-swept laser could be >100 kHz. However, since the mode spacing of the ultrashort cavity can be a few gigahertz, random mode hopping in the cavity leads to unpredictable large distortion in the sweep trace of the swept signal [33]. MEMS-VCSEL and VT-DBR can avoid mode hopping and realize high speed swept signals [3,23,30,34,35,36,37,38], but there are still issues such as high driving voltage, nonlinear sweeping, low output power and complex driving signals. Huber et al. proposed the Fourier domain mode-locked (FDML) laser, which uses a long intracavity fiber delay line to store the whole swept signal to avoid laser buildup from spontaneous emissions [39]. The invention of the FDML laser successfully increases the sweep rate of swept lasers from tens of kilohertz to hundreds of kilohertz and even several megahertz if frequency multiplexing is adopted [40,41,42,43,44,45,46,47], which has attracted much attention in the study of FDML laser and its applications [5,19,47,48,49,50,51,52,53,54,55,56,57,58,59,60].

The principle of the FDML laser is radically different from that of either a traditional continuous wave (CW) laser or a mode-locked laser. The operation of a CW laser depends on the spectral constraints to the signal by a narrow bandpass filter, while a mode-locked laser is realized by applying temporal constraints to the signal by either a modulator or a saturable absorber. In an FDML laser cavity, the swept filter partially constrains the laser signal in the frequency domain, as well as the time domain. The cavity dynamics of FDML lasers are much more complicated than that of either the CW lasers or the mode-locked lasers. To date, a great deal of theoretical modeling and investigation on the laser dynamics in FDML laser cavities, including high-frequency fluctuations on the waveforms [48,49,50,51,52,53], multiple physical effects such as the dispersion and nonlinearity of fiber, linewidth enhancement factor and gain saturation for the instability, and discretization of the FDML laser signals, have been demonstrated [49,61,62,63,64,65,66]. Besides the theoretical investigation to understand the intracavity dynamics, many research groups worldwide have carried out extensive experimental work to enhance the performances of the FDML laser, including the sweep rate [56,67,68,69,70], sweep range [71,72,73,74], coherence length [75,76,77,78,79,80], central wavelength [45,47,77], output power [81,82,83] and stability [66,84,85]. The FDML lasers have been successfully utilized in their application in swept-source OCT systems with micrometer-scale resolution, fiber sensing such as fiber Bragg grating (FBG) configuration, high-speed vibration measurement and refractive index measurement, microwave generation, picosecond pulse generation and spectroscopy systems.

This paper will discuss the recent development of the FDML laser and its applications. Section 2 will introduce the basic principle and different theoretical models together with the intrinsic dynamics of FDML lasers. Section 3 will review and summarize various techniques that could enhance the performance of FDML lasers. Section 4 will present the applications of FDML lasers. Finally, Section 5 discusses the challenges and further development of FDML lasers.

## 2. Theoretical Investigation of FDML Lasers

### 2.1. The Principle of FDML Laser

**Figure 1:**
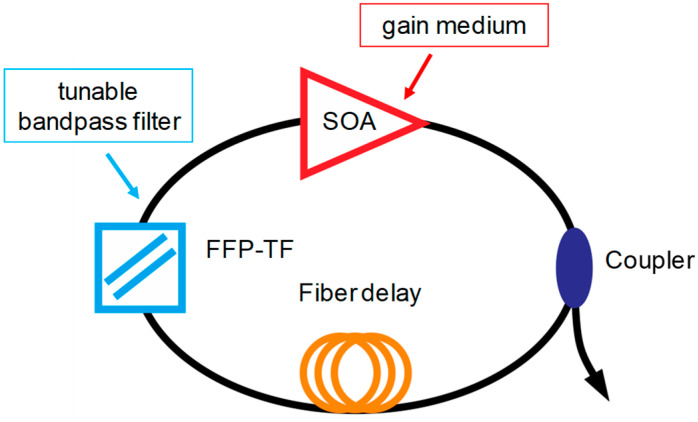
Schematic of a typical FDML fiber laser. The three main elements are the gain, tunable bandpass filter and fiber delay.

Figure 1 shows the schematic of a typical FDML laser. A gain medium with inhomogeneous line broadening, e.g., semiconductor optical amplifier (SOA), is used to amplify the signal. A tunable bandpass filter, either a Fabry–Pérot tunable filter or polygon scanner-based tunable filter, is driven by a periodic electrical signal to set the transient wavelength. A long optical delay that consists of a single-mode fiber, dispersion shift fiber or a combination of single-mode fiber and dispersion compensation fiber is inserted in the FDML laser cavity to buffer the whole swept signal. The basic principle of FDML lasers is that the driving frequency of the tunable filter is synchronized to the cavity round-trip time, which is given by

(1) $$f_{s}=\frac{c}{nl_{c}},$$

where *f*_s_ is the frequency of the sinusoidal signal that drives the tunable filter, *n* is the effective refractive index of the optical fiber, *l*_c_ is the total length of the cavity including the length of the delay fiber and other components and *c* is the speed of light in vacuum.

**Figure 2:**
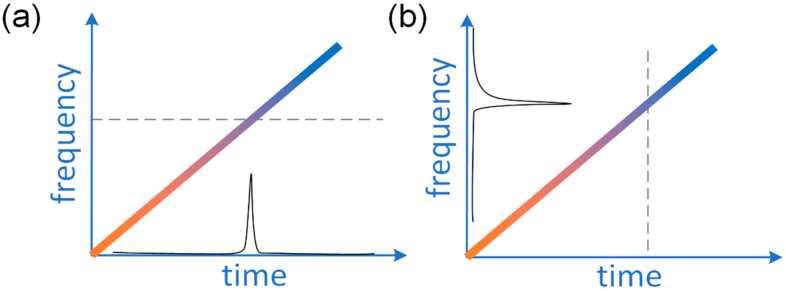
The operation principle of an FDML fiber laser from the viewpoint of (**a**) a single wavelength and (**b**) a given time.

In an FDML laser, a signal with a given carrier wavelength passes through the filter when the central wavelength of the filter is tuned to the targeted wavelength. After passing through the filter, the laser signal will propagate in the laser cavity and reach the filter again when the transmission wavelength of the filter is tuned back to the same wavelength. Thus, the swept signal will pass through the filter without significant loss and does not need to build up from the ASE, which means all modes are simultaneously stored in the long optical delay in the cavity. Therefore, at a given temporal position, the swept filter acts as a quasi-static spectral filter, as shown in Figure 2a, which will filter the spectrum and enhance the temporal coherency of the signal. The modulations for different wavelengths are applied at different times in an FDML laser. The principle of an FDML laser can also be understood by considering a single wavelength as shown in Figure 2b. The signal of a given wavelength inside the sweep range is blocked most of the time except when the filter is tuned to that wavelength. When the central wavelength of the filter passes the given wavelength again, an equivalent modulation will be applied to the signal. The modulation expands the spectrum and generates sidebands to build phase relationships between adjacent cavity modes. The central wavelength of the spectral filtering and the temporal position of the intensity modulation are mapped to each other by the sweep trace of the filter.

### 2.2. Theoretical Models and the Intrinsic Dynamics of Continuous FDML Laser

The first difficulty researchers met in the modeling of FDML lasers is the large time-bandwidth product of ~10^8^ of the swept signal. The propagation of such signals in the kilometer-long fiber is difficult to simulate by the split-step Fourier method commonly used to numerically solve the nonlinear Schrödinger equation. It is also difficult to model the swept filter in the laboratory frame. In 2009, Jirauschek et al. proposed to transfer the modeling of the FDML laser into a reference frame co-moving with the swept filter to reduce the effective time-bandwidth product [62]. The impacts of multiple physical effects, including the dispersion and nonlinearity of the fiber, linewidth enhancement factor and gain saturation, have been investigated using this model [63]. Although we could observe the high-frequency fluctuations on the temporal waveforms in simulations with the co-moving frame model, it could not provide the information required to fully understand the FDML laser dynamics. Slepneva et al. discussed the stability of the steady-state solutions by using a delay differential equation (DDE) [50]. To further understand the intrinsic features of the FDML lasers with long fiber cavity, we simplified the Ginzburg Landau equation (GLE) by dropping the linewidth enhancement factor of the SOA and neglecting the fiber nonlinearity since the power is low. The model can then be reduced to the real Ginzburg–Landau equation (RGLE) with a perturbation term caused by the fiber dispersion [48,53]. These three theoretical models and the intrinsic dynamics of the FDML laser they described will be discussed in detail in this Section.

#### 2.2.1. Co-Moving Reference Frame Model

Unlike conventional mode-locked fiber lasers, it is difficult to numerically simulate FDML lasers because of the large time-bandwidth of >10^8^ induced by the >10 THz spectral range and >10 μs round trip time of the kilometers-long fiber cavity. A theoretical model based on a co-moving reference frame is proposed to reduce the computational requirement [62]. In the laboratory reference frame, the dynamics of the FDML laser could be described with a generalized nonlinear Schrödinger equation (NLSE) based on the slowly varying envelope approximation,

(2) $$\partial_{z}A=\left[g\left(z,i\partial_{t}\right)\left(1-i\alpha \right)-a\left(z,i\partial_{t}\right)-iD_{2}\left(z\right)\partial_{t}^{2}+D_{3}\left(z\right)\partial_{t}^{3}+i\gamma \left(z\right){\left|A\right|}^{2}\right]A,$$

where *A* is the complex amplitude of the envelope of the optical field, *t* is the retarded time, *z* is the position along the propagation direction, *i* is the imaginary number, ∂*_t_* and ∂*_z_* are the partial derivatives with respect to time and distance, respectively, *D*_2_, *D*_3_ and *γ* are the coefficients of the second and third dispersion and fiber nonlinearity, respectively, *g*(*ω*) and *α* are the gain coefficient and linewidth enhancement factor of the SOA, respectively, while *a* describes the linear loss of the cavity and the tunable filter. The tunable filter is periodically driven at frequency *f*_c_ = 1/*T*, where *T* is the cavity roundtrip time. If Equation (2) is solved numerically by the split-step Fourier method directly, the signal covering the full round trip time and the sweep range will require >10^8^ sampling points, which is difficult to be handled in simulations. Another hurdle to the simulation of Equation (2) is the simulation of the tunable filter. The action of the fast time-varying spectral filter could not be modeled by a simple transfer function or in differential form in either the frequency domain or time domain in the laboratory reference frame. However, since the intracavity signal in FDML lasers will only appear in a small spectral range at any given time, i.e., the signal is sparse in the time-frequency space, the simulation could be simplified by changing the frame of reference. Note that the swept filter will become a static filter in a reference frame co-moving with the swept filter. The optical signal *u*(*t*) in the co-moving reference frame is given by

(3) $$u\left(t\right)=A\exp\left(i\int_{t}\omega_{0}\left(t^{\prime }\right)dt^{\prime }\right),$$

where *ω*_0_ is the center frequency of the swept filter. Inserting Equation (3) into Equation (2) and neglecting the high order terms which are assumed to be small, a simplified equation governing the dynamics of the FDML laser is given by

(4) $$\partial_{z}u=\left[g\left(\omega_{0}\right)\left(1-i\alpha \right)-a\left(\omega_{0}\right)+iD_{2}\omega_{0}^{2}+iD_{3}\omega_{0}^{3}+i\gamma {\left|u\right|}^{2}-iD_{2}\partial_{t}^{2}-a_{s}\left(i\partial_{t}\right)\right]u,$$

where *g*(*ω*_0_) and *a*(*ω*_0_) are dynamically evaluated at the instantaneous center frequency *ω*_0_(*t*) of the tunable filter. In Equation (4), the contribution of dispersion is modeled by two linear terms and the action of the tunable filter is modeled by a static transfer function.

Numerical simulations of Equation (4) indicate that the gain, loss, dispersion, nonlinearity of fiber and gain-related linewidth enhancement jointly affect the instantaneous linewidth and shape, coherence length and noise of the FDML laser output. From the input and output signals of each element in the cavity, the contribution of each component of the laser could be studied by simulations to understand the physical mechanism inside the FDML laser cavity [63]. The linewidth enhancement and recovery dynamics in the SOA red shift the power spectral peak. The self-phase modulation induces spectral broadening without any frequency shift. The sweep filter reduces the frequency shift caused by linewidth enhancement and dispersion and simultaneously narrows the linewidth. The fiber dispersion and linewidth enhancement factor of the SOA both contribute to the frequency detuning. The time-dependent frequency detuning will cause filtering loss and destabilize the signal if the frequency detuning is large. The destabilization and suppression of the intracavity signal are counteracted by the rebuilding of signals from the sidebands and noise. The continuous suppression and rebuilding of the optical signal greatly reduce the coherence of the swept signal in the cavity. The instantaneous linewidth of the output signal will be increased to the swept filter linewidth, which is typically at the level of ~10 GHz.

The co-moving reference frame with the swept filter model has been used to study the dispersion, coherence, noise, instantaneous linewidth and polarization of the FDML laser. In 2020, Mark Schmidt et.al. studied the self-mechanism of ultra-stable FDML laser using the co-moving reference frame with the swept filter model. The low noise (sweet-spot) operation can be realized with a small range of residual dispersion [84]. When the residual cavity dispersion exceeds the threshold, it will destroy the sweet-spot operation and induce deep and narrow dips (also referred to as Nozaki–Bekki holes [86]) on the waveform. The number of holes in the intensity trace will increase when the residual dispersion in the FDML laser cavity increases. The evolution of the instantaneous frequency with different amounts of dispersion with or without group delay is further analyzed, which indicates that the accumulated chirp per roundtrip can be compensated by the group delay of the tunable filter [66].

#### 2.2.2. Delay Differential Equation Model

In 2013, Slepneva et al. discussed the stability of the steady-state solutions by using a set of delay differential equations (DDE) [50], which are

(5) $$\partial_{t}A+A-i\Delta \left(t\right)A=\sqrt{\kappa }\exp\left[\left(1-i\alpha \right)G\left(t-T\right)/2\right]A\left(t-T\right),$$

(6) $$\partial_{t}G=\gamma \left[g_{0}-G-\left(e^{G}-1\right){\left|A\right|}^{2}\right],$$

where *A*(*t*) is the electric field envelope at the input of the SOA and the carrier density is modeled with a saturable gain *G*(*t*). Δ(*t*) describes the swept filter. *Κ* is the linear loss factor per round trip. *g*_0_, *α* and *γ* are the linear unsaturated gain, linewidth enhancement factor and normalized gain relaxation rate, respectively, in the SOA. Equation (5) describes the effect of the swept filter and includes the amplification of the signal in the SOA. Equation (6) describes the gain saturation of the SOA. Although the DDE does not include the signal distortion introduced by the propagation in a dispersive and nonlinear fiber, it is effective in the investigation of the instability of the stationary signal in FDML lasers induced by the linewidth enhancement factor of SOA and the relative frequency shift caused by the cavity dispersion or sweep rate detuning of the tunable filter. In the numerical simulations of the DDE, both modulational and Turing instabilities are observed in the dynamics of FDML lasers when the relative frequency shift exceeds the thresholds. Increasing the linewidth enhancement factor of the SOA will affect the boundary of the instabilities differently on the red and blue detuning sides [50]. For large *α*, the Turing instability will be triggered on the red detuning side and modulational instability will dominate on the blue detuning side [50].

#### 2.2.3. Perturbed Real Ginzburg–Landau Equation Model

The periodically driven swept filter is the only component that differentiates an FDML laser from other types of lasers. To obtain a broad sweep range, an inhomogeneous or fast recovery saturable gain should be included. Other effects such as dispersion, nonlinearity and linewidth enhancement factor are typically found in FDML laser cavities, but they are not critical to the building of an FDML laser. Investigations with Equation (4) by turning on and off specific physical effects in the simulations provide some understanding of the impact of a particular physical effect on the FDMP laser output. However, these studies are phenomenological and cannot provide an in-depth understanding of the laser dynamics. Specifically, Equation (4) cannot be used to investigate the nature of the high-frequency fluctuations on the FDML laser output waveforms. Understanding the nature of these high-frequency fluctuations is the first step in their control and reduction which would improve the performance of FMDL lasers. To understand the intrinsic dynamics of FDML lasers, the contribution of the various physical effects should be properly ordered according to their impact. By utilizing a Wentzel–Kramers–Brillouin (WKB) analysis of Equation (2) with frame transformation of Equation (3), we find the nonlinear phase shift and the in-band dispersion both have smaller contributions that can be neglected in the first-order approximation. Then, Equation (2) is reduced to

(7) $$\partial_{z}u=g(u,\omega_{0})(1-i\alpha )u-\sigma (\omega_{0})u-a\left(i\partial_{t}\right)u+iD_{2}\omega_{0}^{2}(t)u+iD_{3}\omega_{0}^{3}(t)u,$$

where *g*(*u*) = *g*_0_/(1 + |*u*|^2^/*I*_sat_) is the saturated gain with negligible recovery time, *I*_sat_ is the saturated power of the gain and σ is the linear loss. It should be noted that such a fast response gain saturation model is not only valid in the case of a fast recovery gain, but also in cavities with a slow recovery gain and a fast response nonlinear loss element, such as a nonlinear optical loop mirror, nonlinear polarization rotation setup and other equivalent components. The simplified model of Equation (7) captures the essential dynamics of the FDML lasers, especially the high-frequency intensity fluctuation on the waveform. Equation (7) can be simplified further by expanding the gain saturation factor in the Taylor series of |*u*|^2^. By keeping the first-order term only, Equation (7) can be reduced to

(8) $$\partial_{z}u=(g_{0}-\sigma )u-\frac{g_{0}}{I_{\mathrm{sat}}}{\left|u\right|}^{2}u+\frac{1}{2B^{2}}\partial_{t}^{2}u+iD_{2}\omega_{0}^{2}(t)u+iD_{3}\omega_{0}^{3}(t)u,$$

where *B* is the filter bandwidth. Equation (8) can be normalized to

(9) $$\partial_{Z}U=U-{\left|U\right|}^{2}U+\partial_{T}^{2}U+iC(T)U,$$

where the normalized variables are defined as

(10) $$\begin{array}{l} U=u\sqrt{g_{0}/\left[2(g_{0}-\sigma )I_{\mathrm{sat}}\right]},\\ Z=z(g_{0}-\sigma ),\\ T=tB\sqrt{2(g_{0}-\sigma )},\\ C(T)=S_{2}\Omega_{0}^{2}(T)+S_{3}\Omega_{0}^{3}(T),\\ \Omega_{0}(T)=\omega_{0}(T)/[B\times \sqrt{2(g_{0}-\sigma )}],\\ S_{2}=D_{2}B^{2}\sqrt{8{\left(g_{0}-\sigma \right)}^{3}I_{\mathrm{sat}}/g_{0}},\\ S_{3}=D_{3}B^{3}4(g_{0}-\sigma {)}^{2}\sqrt{I_{\mathrm{sat}}/g_{0}}, \end{array}$$

where *U*, *Z* and *T* are the normalized amplitude, propagation distance and time, *C*(*T*) describes the dispersion-induced frequency shift, which has included the normalized sweep trace Ω_0_(*T*) and the normalized dispersion coefficients *S*_2_ and *S*_3_. Equation (9) is a modified RGLE with a perturbation term *iC*(*T*)*U*. In an ideal cavity without dispersion, Equation (9) will be reduced to a standard RGLE as

(11) $$\partial_{Z}U=U-{\left|U\right|}^{2}U+\partial_{T}^{2}U.$$

The RGLE in the co-moving reference frame has included the essential terms to capture the fundamental dynamics of an FDML laser, i.e., a fast recovery saturable gain and a periodically driven swept filter, which is modeled as a static filter in the co-moving frame.

The RGLE has been extensively studied in fluid dynamics. The RGLE has stationary solutions which are single frequency continuous waves in our system. The stationary solution with normalized angular frequency Ω is

(12) $$U\left(Z,T\right)=\sqrt{1-\Omega^{2}}\exp\left[i(KZ-\Omega T)\right].$$

From Equation (12), there are stationary solutions in the frequency region |Ω| < 1. However, the stationary solutions are unstable when Ω^2^ > 1/3—which is known as Eckhaus instability.

In FDML lasers with non-zero cavity dispersion, the cavity dispersion will perturb the RGLE as described by Equation (9). Equation (9) does not have stationary solutions. By using multi-scale analysis, the zero-th order equation is the Riccati equation, which has the evolutionary solution ${\bar{U}}^{0}$ given by

(13) $${\bar{U}}^{0}(Z)=\frac{\exp\left[-i{\bar{\Omega }}_{0}\left(t_{2}\right)t_{1}\right]}{\sqrt{I_{0}^{-1}\exp\left[Q(0)-Q(Z)\right]+2\int_{0}^{Z}\exp\left[Q(x)-Q(Z)\right]dx}},$$

where *I*_0_ and ${\bar{\Omega }}_{0}(t_{2})$ are the initial intensity and frequency of ${\bar{U}}^{0}$ at *Z* = 0, $Q(Z)=\int_{0}^{Z}R(x)dx$ and $R(Z)=2\left[1-{\left({\bar{\Omega }}_{0}(t_{2})-C^{\prime }Z\right)}^{2}\right]$. The stability condition of the solution in Equation (13) is

(14) $$\lambda_{\max}=2{\left(C^{\prime }Z-{\bar{\Omega }}_{0}\right)}^{2}-{\left\{I_{0}^{-1}\exp\left[Q(0)-Q(Z)\right]+2\int_{0}^{Z}\exp\left[Q(x)-Q(Z)\right]dx\right\}}^{-1}<0.$$

The condition Equation (14) will be reduced to the Eckhaus criterion when *C*′ = 0. In an FDML laser, since *C*′ is a slowly varying function of wavelength, different time points on the temporal waveform, which have different instantaneous wavelengths, will violate the stability criterion after different numbers of round trips. At a given round trip *Z*, high-frequency fluctuations will appear at the boundaries between the stable and unstable regions. The continuous triggering of the Eckhaus instability will eventually fill the whole waveform with the high-frequency fluctuations, which will significantly degrade the signal quality and increase the instantaneous linewidth [48]. It should be noted that when nonlinear phase terms such as the linewidth enhancement factor are included, the Eckhaus instability will be replaced by a more general form, i.e., the Benjamin–Feir instability.

### 2.3. Theoretical Investigation of Discrete FDML Lasers

Since the swept filter only confines the intracavity signals partially in the frequency and time domains, the signals at different wavelengths and temporal points are not strongly related, which results in low coherence and low signal quality. A straightforward approach to improve the coherence of the swept signal is to strengthen the constraints applied to the signal, either in the time domain or frequency domain, or a combination of both. It has been experimentally demonstrated that inserting a comb filter into the FDML laser cavity to discretize the swept signal will enhance the coherence length of the swept signal [79,80]. Adopting a microring comb filter in the cavity has successfully improved the coherence length of an FDML laser to >100 mm [80], which is an order of magnitude longer than that of conventional FDML lasers. Discretization of the FDML laser can also be realized in the time domain by applying a sequence of pulsed intensity modulation on the intracavity signal. Time-domain discretization does not improve the coherence length but has been shown to significantly improve the stability of the temporal waveforms in experiments [87]. However, the dynamics of discrete FDML lasers have not been systematically investigated in theory, although some preliminary modeling has been carried out.

Modeling of an FDML laser discretized in the time domain is rather simple since intensity modulation can be modeled by adding a varying attenuation term into Equation (4). Preliminary simulations show that a properly chosen short-pulse modulation could improve the signal quality in both the time and frequency domains [88]. However, the simulation focused on a single pulse. A systematic investigation is required to understand the full dynamics and the potential to improve the signal quality in the full sweep range. Compared with time-domain discretization, modeling an FDML laser discretized in the frequency domain is more difficult because the swept filter and the static comb filter could not be modeled in the same reference frame. In the numerical simulations, we proposed to simulate the effect of the static comb filter in the laboratory frame and the swept filter in the co-moving frame. In this dual-frame model, a pair of conjugate frame conversions is applied to the signal in each round trip [89]. Simulations of frequency domain discretization by an F-P comb filter using the dual-frame model successfully demonstrate improvement in the signal quality [89]. However, numerically simulating the full sweep range of >10 THz remains challenging because of the large number of sampling points required. Adaptive algorithms should be adopted together with the dual-frame model in future investigations of discrete FDML lasers.

## 3. Experimental Techniques for High-Performance FDML Laser

### 3.1. High Sweep Rate

The FDML laser uses a long fiber as the delay in the cavity to store the whole frequency sweep and avoids the rebuilding of the laser signals from spontaneous emission. In principle, the sweep rate is only limited by the speed of the tunable filter. Currently, a commercially available tunable filter with more than 100 nm free spectral range can achieve a tuning speed of 170 kHz. A custom-built tunable filter can achieve a tuning speed of 419 kHz. Therefore, the fundamental sweep rate of the FDML laser can reach 419 kHz [90]. The sweep rate of an FDML laser can be increased further by either harmonic mode-locking which drives the tunable filter at the *n*-th, *n* = 2, 3, 4, …, resonance frequency [91,92] or buffering which copies and delays the sweeps [42,47,68,69,70,93,94].

**Figure 3:**
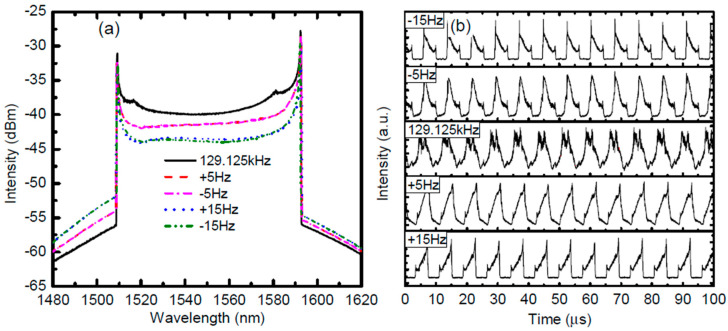
The (**a**) integrated spectra and (**b**) waveforms of the FDML fiber laser harmonically mode-locked in the third order. The scan frequency of FFP-TF is 129.125 kHz and relative detunes are ±5 Hz and ±15 Hz. Reprinted with permission from Ref. [92].

In an FDML laser, the tunable filter can be driven at either the fundamental frequency or one of the harmonics of the fundamental frequency of the laser cavity. If the tunable filter is driven at the *n*-th harmonic frequency, the sweep rate of the FDML laser is increased to *n* times that of driven at the fundamental frequency. An FDML laser running at the third harmonic frequency has been realized to increase the fundamental sweep rate of the swept signal [91,92]. In addition to increasing the sweep rate, driving the tunable filter at the third harmonic frequency of an FDML laser will provide higher flexibility in the manipulation of multiple sweeps, which has been demonstrated in discrete FDML lasers with time-domain modulation [95]. Harmonically FDML laser also enables the observation of super-mode noise peaks in the RF spectrum as shown in Figure 3. The signal-to-noise ratio is related to the detune. When the sweeping frequency is detuned from the harmonic frequency of the FDML laser, the signal-to-noise ratio decreases, which helps to study the detune between the swept signal and the third harmonics sweep trace of the tunable filter [92,96]. The main problem for a harmonically FDML laser is that the sweep range is narrow because the amplitude response of the high-frequency driving for the tunable filter is low.

**Figure 4:**
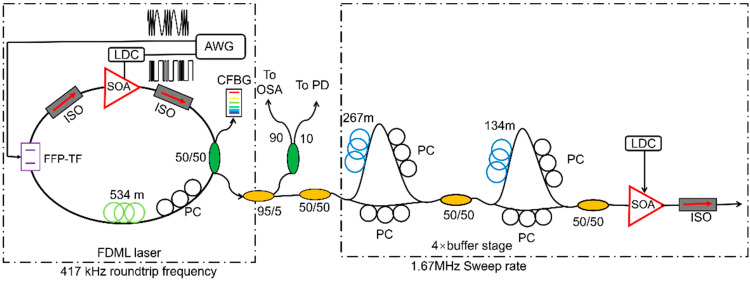
Schematic of an FDML laser with an optional buffer stage: AWG, arbitrary waveform generator; LCD, laser diode controller; SOA, semiconductor optical amplifier; ISO, optical isolator; PC, polarization controller; BFP-TF, Fabry–Pérot filter; OSA, optical spectrum analyzer; PD, photo diode. Adapted with permission from Ref. [42].

The sweep buffering technique can be used to further increase the sweep speed, which combines a series of optical splitters and couplers with fiber delay lines in one arm of a Mach–Zehnder interferometer. The additional length of the fiber can be one-half, one-fourth or one-eighth of the optical length of the FDML laser, which duplicates sweep copies by splitting and recombination to increase the sweep rate as shown in Figure 4. The sweep buffering technique is widely used in FDML lasers to realize a 4-fold, 8-fold or even 16-fold increase in the sweeping speed to several hundred kilohertz or even megahertz [14,42,56,58,67,68,69,70,94]. The phase relation between different copies of the swept signal is stable and therefore ideal for applications in phase-sensitive and Doppler OCT imaging systems. In addition, the effective duty cycle of the swept signal can be improved with multiple buffering stages. However, the buffering technique suffers from the insertion loss of the couplers and fibers, which will decrease the sensitivity when the FDML lasers are used in imaging. The problem can be mitigated by adding an extra SOA to compensate for the loss or utilizing polarization splitters and combiners. The birefringence effects in the long fiber should be controlled by adding a polarization controller in one arm of the Mach–Zehnder interferometer.

### 3.2. Broadband Sweep Range

**Figure 5:**
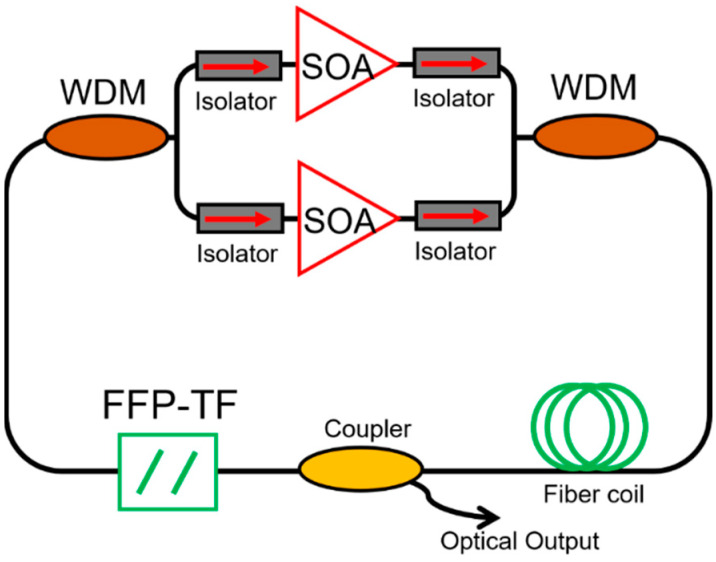
Schematic of a broadband FDML laser which consists of two SOAs of adjacent wavelength bands arranged in parallel. Isolators are used before and after each SOA to ensure unidirectional operation. The SOAs are then coupled through two WDMs to complete the ring-cavity. Other broadband components include an FFP-TF for wavelength tuning, a fiber coil to set the cavity resonance frequency and a fiber coupler for output coupling. Adapted with permission from Ref. [73].

Broadband swept sources can improve the axial resolution in swept-source OCT imaging systems, and accuracy in chemical spectroscopy and optical sensing. To achieve a broadband sweep range, booster SOA with wide gain bandwidth has been used in an FDML laser to generate up to 143 nm wide spectrum [42]. Combining two SOAs in parallel has also been used in FDML lasers to increase the sweep range. The first demonstration was carried out by using two SOAs at the central wavelength of 1272 and 1322 nm, respectively, in an FDML laser, which generated a 145 nm sweep range [97]. A similar method was later used to increase the sweep range to 160 nm [71]. To further improve the sweep range, Kevin et al. proposed to use two SOAs at the central wavelength of 1.3 and 1.5 µm together with two wavelength-division multiplexers (WDMs), as shown in Figure 5. A sweep range of 284 nm is obtained, which is the broadest sweep range reported to date [73]. The FDML laser was applied in an OCT system and achieved a 7 µm axial resolution. Using two SOAs in parallel typically suffers from the presence of a sweep gap which can be several nanometers wide. The sweep gap can be minimized by choosing the appropriate central wavelengths and gaining the bandwidth of the SOAs. To increase the sweep range further, the free spectral range of the tunable filter should be improved to avoid spectral overlap.

### 3.3. High Output Power

Output power is another key parameter for FDML lasers. The direct method to increase the laser output power without affecting the sweep range is to increase the gain of the SOA in the laser or to incorporate booster SOAs in the laser cavity. External booster SOAs are often used before the swept signal is injected into the sample in the OCT system to ensure a sufficient intensity level for imaging and detection [45,98]. Booster SOAs can provide 100 nm gain bandwidth and nearly 30 dB gain, which meets the requirement for most OCT imaging systems. External amplification has a more complex structure and higher cost because of the additional SOAs. The output spectrum also often suffers from modulations because the booster SOA is sensitive to polarization.

**Figure 6:**
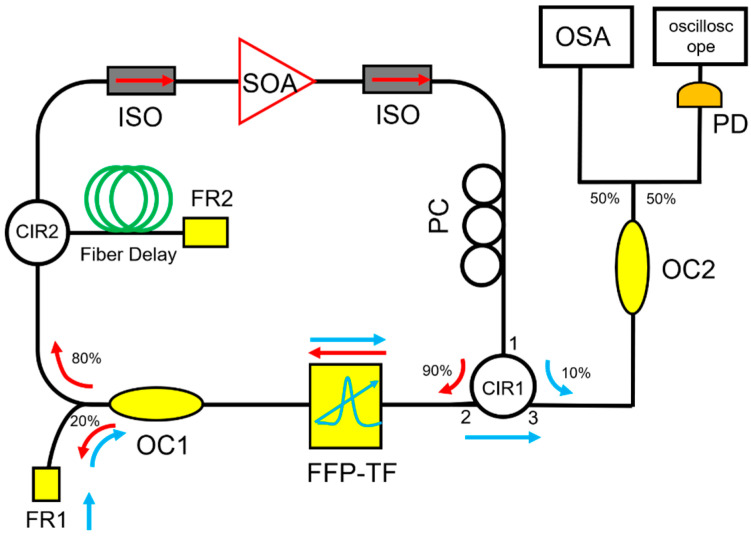
Schematic of an FDML laser that reuses the SOA in the laser cavity. SOA, semiconductor optical amplifier; ISO, isolator; PC, polarization controller; FFP-TF, fiber Fabry–Pérot tunable filter; OC, optical coupler; CIR, circulator; FR1 and FR2, Faraday mirror. Adapted with permission from Ref. [82].

Other amplification schemes using gain media such as Raman amplifier [74,83,99,100,101,102], Erbium-doped fiber amplifier [40,103] and Ytterbium-doped fiber amplifier [104] in the laser cavity can also increase the output power of FDML lasers. Combinations of rare-earth-doped gain or Raman gain have also been demonstrated for high output power [100]. Besides changing the gain medium, the output signal of the FDML laser can be further amplified by using a master oscillator power amplification (MOPA) configuration and the power can be increased up to a 2000 mW output power [105]. However, it is difficult to obtain a broad spectrum in FDML lasers using rare-earth-doped gain because their gain recovery time is at a sub-millisecond level, which is much longer than the whole sweep period of an FDML laser. On the other hand, the linewidth enhancement factor α of SOA is one of the major effects that degrade the signal quality in amplification, when compared to rare-earth-doped fiber amplifiers. Instead of changing the gain medium, the configuration of the FDML laser can also be modified to increase the output power. In 2021, Zhiwei Yang et al. proposed and demonstrated a novel amplification scheme that reuses the SOA in the laser cavity as shown in Figure 6 [82]. The SOA is used as the ASE in the cavity and provides the first amplification stage, which increases the output power. This technique, with its simple structure and broadband gain bandwidth, can increase the output power compared with traditional FDML laser while maintaining a more than 100 nm wide sweep range. This intra-cavity amplification scheme will also be used to modify the spectral shapes.

### 3.4. Long Coherence Length

Although FDML lasers have superior performance in both the sweep rate and sweep range, they do not show significant improvement in the coherence length when compared with other short cavity swept lasers. This is puzzling because FDML lasers are expected to output highly coherent swept signals as the rebuilding of laser signals is avoided by design. The swept signals in FDML lasers in general show dense disordered high-frequency fluctuations on the waveforms, which degrade the signal quality and lower the coherency. In 2012, Biedermann et al. theoretically showed that the fiber dispersion, nonlinearity and linewidth enhancement factors of the SOA jointly deteriorate the sweep trace of the tunable filter and induce a time-dependent relative frequency shift, which deteriorates the signal and reduces the coherence length of the FDML lasers [63]. It has been shown that if the optical signal in an FDML laser accumulates sufficient frequency shift, the Eckhaus instability will be triggered, which will lead to high-frequency fluctuations in the optical signals [48]. New sidebands are also generated and amplified, which will push the optical signal away from the center wavelength of the filter in subsequent round trips. The instantaneous linewidth is broadened because of the high-frequency instabilities on the waveforms. Stable output without the high-frequency fluctuations clearly is one of the desirable features of the next generation FDML lasers.

#### 3.4.1. Dispersion Compensation

Because of the chromatic dispersion in the long fiber delay line, the synchronization between the optical roundtrip time of the signal and the central wavelength of the tunable filter can only be satisfied at a specific wavelength, thus degrading the coherence of the FDML laser output. Even an FDML laser operating at the central wavelength of 1310 nm, which is the zero-dispersion point of the single-mode fiber, suffers from mismatch for the sweep trace of the tunable filter when the sweep range is at 100 nm level. When the FDML laser is operating at the 1550 nm wavelength region, the mismatch induced by dispersion is given by

(15) $$\partial_{z}u=\left[iD_{2}\omega_{0}^{2}+iD_{3}\omega_{0}^{3}-iD_{2}\partial_{t}^{2}\right]u.$$

Then the signal propagation in the fiber delay could be presented by a single transfer function as

(16) $$u_{\mathrm{out}}(t)=u_{\mathrm{in}}(t)\exp\left\{i\left[D_{2}\omega_{0}^{2}(t)+D_{3}\omega_{0}^{3}(t)\right]L\right\}.$$

Since *ω*_0_(*t*) varies periodically with time, the optical signal will experience a time-dependent shift in the instantaneous frequency as

(17) $$\Delta \omega (t)=-\frac{\partial \phi }{\partial t}=\frac{d\omega_{0}(t)}{dt}\times \left[2D_{2}\omega_{0}(t)+3D_{3}\omega_{0}^{2}(t)\right]L.$$

Consider an FDML laser with a filter sweeping sinusoidally in a range of 2 *B*_r_ of 10 THz and frequency *f*_s_ of 50 kHz, i.e., *ω*_0_(*t*) = *B*_r_ sin(2π*f*_s_*t* + *ϕ*_0_), *B*_r_ *=* 10π × 10^12^ s^−1^, and *f*_s_ = 5 × 10^4^ Hz, and *ϕ*_0_ is the initial phase of *ω*_0_(*t*). The frequency shift of Equation (17) becomes

(18) $$\Delta \omega (t)=\pi f_{s}B_{r}{}^{2}L\times \left[2D_{2}+3D_{3}B_{r}\sin(2\pi f_{s}t)\right]\sin(4\pi f_{s}t).$$

We then use *D*_2_ = −8.5 ps^2^/km and *D*_3_ = 0.02 ps^3^/km, which are the dispersion coefficients at ~1550 nm for SMF-28e single-mode optical fiber, to estimate the magnitude of the frequency shift. After propagation in 4 km SMF in the cavity to match the sweep rate of 50 kHz, the frequency shift will be

(19) $$\Delta f(t)=\Delta \omega (t)/2\pi =\pi^{2}\times 10^{7}\times \left[0.6\pi \sin(2\pi f_{s}t)-17\right]\times \sin(4\pi f_{s}t).$$

**Figure 7:**
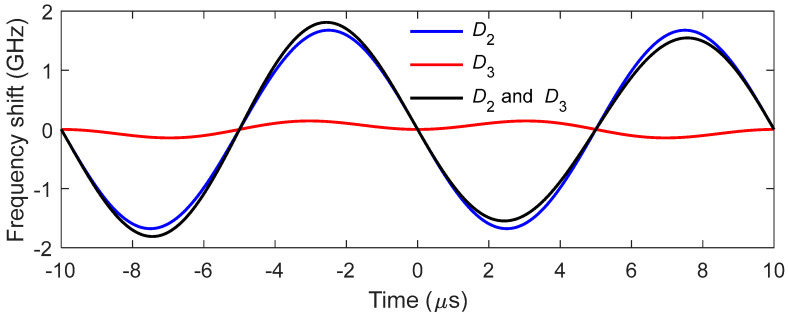
Relative frequency shift induced by dispersion after propagation in 4 km SMF. The blue, red and black solid curves represent the frequency shifts caused by *D*_2_, *D*_3_ and their combined effect, respectively.

Figure 7 shows the frequency shifts caused by *D*_2_ (blue solid curves), *D*_3_ (red solid curves) and the combined effect of *D*_2_ and *D*_3_ (black solid curves). It should be noted that the frequency shift of Equation (19) is defined in the filter reference frame, the frequency shift is the relative frequency offset between the carrier frequency of the optical signal and the center of the filter passband induced by the dispersion after propagation of one round trip in the cavity. From Figure 7, the dispersion-induced frequency shift could be larger than 1.8 GHz in just a single round trip. As the typical linewidth of the tunable filter is 10 GHz, the dispersion induced frequency shift will push the signal out of the passband of the tunable filter in just a few round trips.

**Figure 8:**
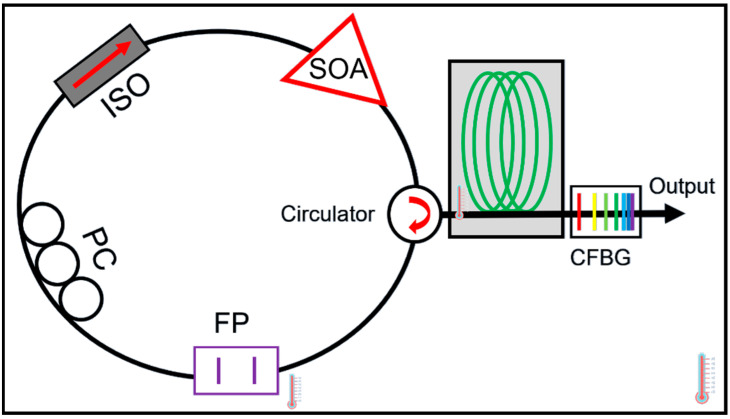
Schematic of an FDML laser with chirped fiber Bragg grating. It comprises a 1310 nm semiconductor optical amplifier (SOA), a fiber polarization controller (PC), a fiber optics isolator (ISO), a fiber Fabry–Pérot filter (FFP) driven at 411 kHz, a mixed fiber spool and a custom made chirped fiber Bragg grating (CFBG). Adapted with permission from Ref. [14].

The cavity dispersion-induced frequency shift is the dominant effect that degrades the signal quality of FDML lasers. To reduce the cavity dispersion, dispersion-shifted fiber (DSF) [13], a combination of single-mode fiber (SMF) and dispersion compensation fiber (DCF) [80,87,106], Corning HI1060 [29,41] and chirped fiber Bragg gratings (CFBG) [42,45,76] have been proposed to improve the coherence length of the FDML lasers. For FDML lasers operating at 1550 nm, the DSF was used to improve the coherence length to 12.8 mm compared with a coherence length of 7.6 mm if single-mode fiber was used [13]. Besides, as DCF is available for the 1550 nm region, a combination of SMF with DCF was also proposed to reduce the dispersion in the FDML laser cavity [80,87,106]. The main problem for DSF and DCF is that it is impossible to completely compensate for the dispersion as the high order dispersion cannot be eliminated. Furthermore, for FDML lasers operating at 1310 nm and 1050 nm, there is no DCF for compensation, so CFBG is often used to eliminate the dispersion [42,45,76]. Pfeiffer et al. demonstrated an ultra-low noise output in >100 nm spectral range with a temperature-control precision of 0.001 °C and a dynamically controlled CFBG to eliminate the residual dispersion at 1310 nm as shown in Figure 8 [14]. For operation at wavelength 1050 nm, HI1060 fiber and CFBG have been utilized in FDML lasers to increase the coherence length [29]. Such stringent requirement for active control by using CFBG makes it difficult to be adopted outside the laboratory. To avoid dispersion, non-dispersion components such as a photonic crystal cavity resonator were proposed in FDML lasers. However, the photonic crystal cavity resonator has a sweep range of only 3.5 nm with large fluctuations [107]. Low loss hollow-core fibers are a promising candidate for use as fiber delay for FDML lasers to completely avoid dispersion and nonlinearity to generate a highly stable and long coherence swept signal.

#### 3.4.2. Frequency Discretization with Comb Filter

The dispersion and nonlinearity of fiber and the linewidth enhancement factor of the SOAs in FDML lasers will degrade the instantaneous linewidth of an FDML laser by generating high-frequency fluctuations on the waveform. Incorporating a Fabry–Pérot comb filter into the conventional FDML laser cavity to discretize the swept signal has been proven effective in improving signal quality and narrowing the instantaneous linewidth [16,79,80,106]. A frequency comb swept signals with equal spacing in the frequency domain are generated with an FP comb filter. Because of the narrow linewidth of the comb filter, the swept signal has narrower instantaneous linewidth which leads to a longer imaging depth in the OCT systems. A comb filter with 0.015 nm linewidth is inserted in the FDML laser and a 1.2 dB sensitivity roll-off over a 3 mm range is realized [79]. The sweep rate and FSR of the comb filter determine the period in the OCT imaging system, for example, a 25 GHz comb line will have a period of 3 mm. To take full advantage of the long coherence length of the swept signal, circular ranging techniques have been used to realize large range imaging with relatively low acquisition bandwidths [108]. In addition, the equally spaced comb lines can be used directly as the clock signal for frequency calibration in the OCT interference fringe acquisition.

**Figure 9:**
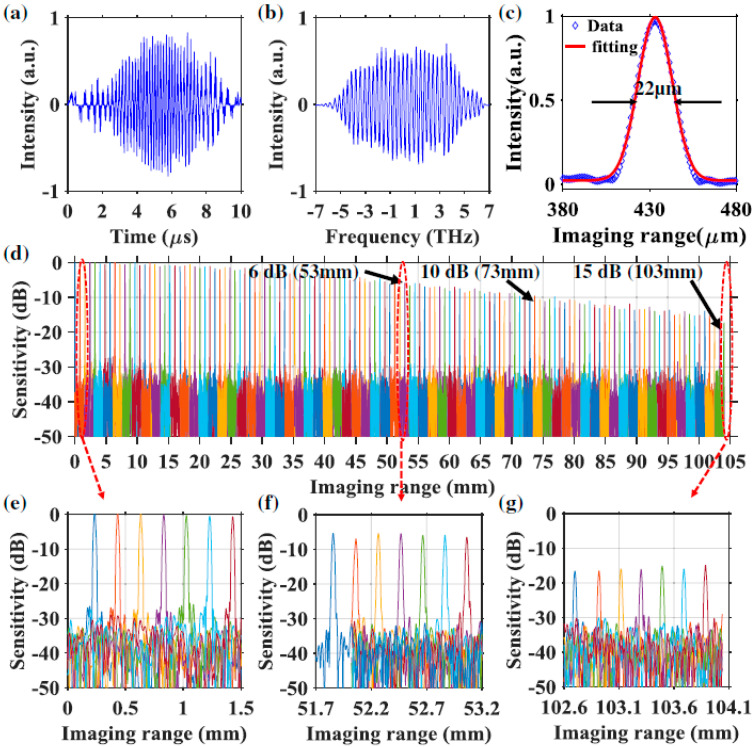
(**a**) Example of raw interference fringe pattern with microring comb filter captured by the BPD. (**b**) Resampled interference spectrum with self-clocking. (**c**) Axial resolution estimation with PSF calculated from the signal of (**b**). The measured PSFs of frequency comb swept laser with microring comb filter for OCT imaging range from (**d**) 0 to 104 mm, (**e**) 0 to 1.5 mm and (**f**) 51.7 to 53.2 mm, covering the 6 dB sensitivity roll-off length. (**g**) The zoom-in view from 102.6 to 104.1 mm, covering the 15 dB sensitivity roll-off length. Reprinted with permission from Ref. [80].

The linewidth of the comb filter is the key factor that determines the instantaneous linewidth of the swept signals. However, it is difficult to reduce the linewidth of Fabry–Pérot comb filters. Compared with Fabry–Pérot comb filters, whispering gallery mode-based microresonators with high Q factor and much narrower linewidth are a better choice as the comb filter for discrete FDML lasers. We have proposed and experimentally demonstrated a highly coherent frequency comb swept laser by discretizing an FDML laser with an on-chip Hydex glass microring filter which has a Q-factor of ~2 × 10^6^ [80]. Benefitting from the high Q factor of the microring filter, the linewidth of the discrete comb lines from the FDML laser is only 1.5 GHz. When these discrete comb lines are applied in an OCT imaging system, the 6-dB sensitivity roll-off length can reach 53 mm. Since the optical path difference is twice that of the roll-off length, the coherence length of the laser is 106 mm, which, to the best of our knowledge, is the first time 100 mm level coherence length is realized in FDML lasers. Furthermore, the 15-dB sensitivity roll-off length extends to more than 100 mm as shown in Figure 9. The ultra-long coherence length obtained in the frequency comb swept laser is due to the narrow linewidth of the microring comb filter, which is consistent with the theoretical simulation. The ultra-long coherence length-frequency comb swept laser will find applications in long-range OCT, including circular interferometric imaging.

### 3.5. High Stability with Low Noise

FDML lasers with high stability and low noise are essential for many applications such as OCT imaging, fiber sensing and LiDAR. In OCT imaging systems, the swept signal from the FDML laser is used as the clock signal. Therefore, fluctuations in the frequency will cause desynchronization leading to a false interpretation of the spectrum. For LiDAR application, each of the swept signals is used to take a distance measurement, thus timing jitter of the FDML lasers will lead to errors in the measurement. To ensure the desired sweet spot operation of FDML lasers with high stability, many methods including temperature control, cavity length control, external feedback and time domain discretization have been proposed and demonstrated.

#### 3.5.1. Control Techniques

The mismatch between the roundtrip time of the light and the driving frequency of the tunable filter will cause instabilities. To reduce the mismatch, the temperature-dependent components such as the fiber delay or CFBG of the FDML laser are placed in a Peltier temperature control unit which has a precision of 0.001 °C [14]. In this case, the temperature-induced frequency detuning is limited to less than ±1 mHz by using an automated temperature regulation, which enables the FDML laser to operate close to the sweet spot region with high stability. Precision control of the temperature can significantly reduce the noise in FDML laser output and facilitates the study of the noise characteristics at different frequency detuning [14]. The intensity noise features at frequency detuning of 0, 1 and 5 Hz are demonstrated, where larger frequency detuning generates a higher noise [14]. The noise intensity can therefore be used in a feedback mechanism to minimize frequency mismatch and generate a highly coherent swept signal in FDML lasers.

**Figure 10:**
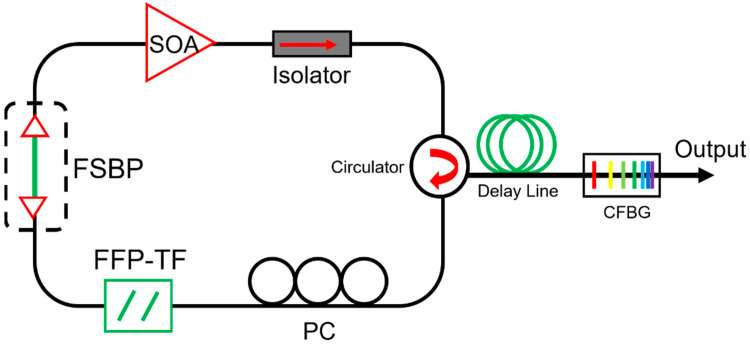
Schematic diagram of an FDML laser with the motorized free space beam path (FSBP). Adapted with permission from Ref. [85].

Besides temperature control, cavity length control is another direct method to reduce the mismatch between the fundamental resonance of the laser cavity and the driving frequency of the tunable filter. Cavity length control has been used in frequency comb lasers to stabilize the repetition rate. To ensure synchronization in FDML lasers, a motorized free space beam path has been inserted in the laser cavity by adjusting the free space beam path by the cavity control module as shown in Figure 10 [85]. The cavity length control algorithm is proposed and can achieve an accuracy of 0.3125 μm.

**Figure 11:**
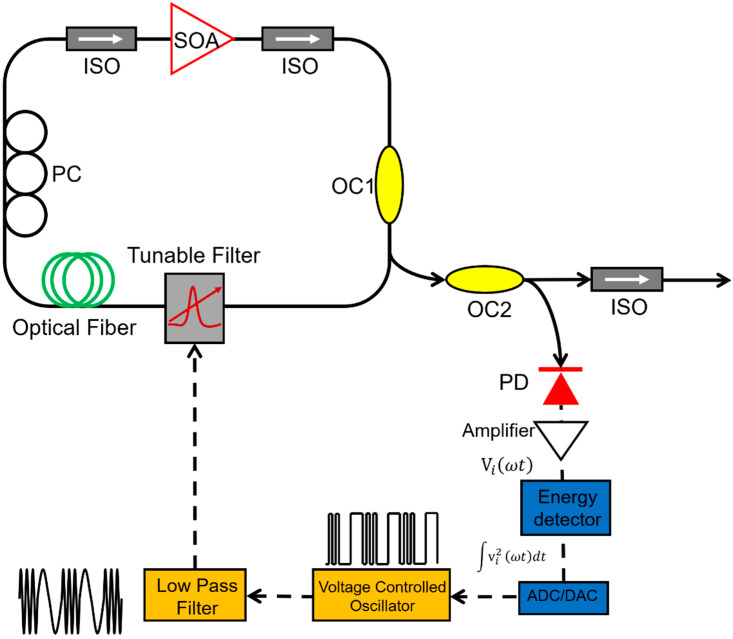
Schematic diagrams of the self-starting, self-regulating FDML laser. Adapted with permission from Ref. [109].

External feedback is a common technique to realize stable output in laser systems. A self-starting and self-regulating FDML laser was proposed in 2011 by Xingde Li et.al. [109]. A voltage-controlled oscillator (VCO) is incorporated into an FDML laser, which uses feedback control to adjust the driving frequency of the tunable filter to maximize the optical energy in the laser cavity as shown in Figure 11. The output power of the FDML laser was maintained at ~95% of the initial level compared to only ~40% in conventional FDML lasers without feedback control. The external feedback-controlled FDML laser reduced the effect of external disturbances such as temperature and humidity on the optical path or frequency jitters of the tunable laser.

#### 3.5.2. Time Domain Discretization

Apart from using a comb filter to discretize the swept signal in an FDML laser, time-domain modulation by an optical modulator can also achieve a discrete swept signal. The main advantage of time-domain modulation is that a highly stable swept signal with low timing jitter can be generated due to the confinement in the time domain. Another advantage of time-domain discretization is that it can realize reconfigurable discrete swept signals with variable comb lines and pulse widths by adjusting the driving pulse patterns on the optical modulator with an arbitrary waveform generator. We proposed and demonstrated time-domain modulation in an FDML laser and applied the reconfigurable discrete swept signal in an OCT imaging system [87,110].

**Figure 12:**
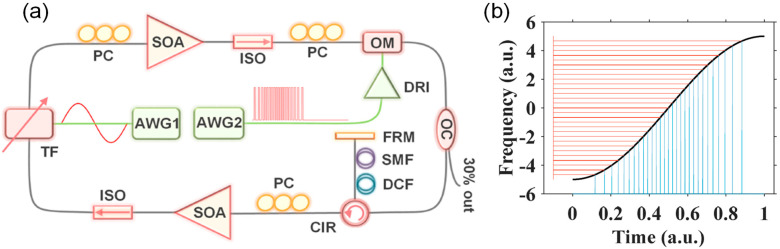
(**a**) Schematic diagram of an FDML laser with time domain modulation and (**b**) the principle of discretization of swept signal with identical comb lines in the frequency domain. The blue solid lines are the rate varying pulses in the time domain and the red solid lines are the corresponding uniform comb lines in the frequency domain. Reprinted with permission from Ref. [87].

**Figure 13:**
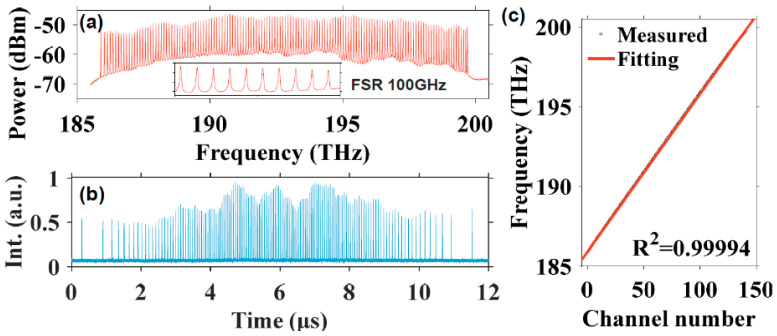
The (**a**) spectrum (the inset is a zoom-in view from 193 to 194 THz) and (**b**) temporal waveform of the discrete FDML laser with 100 GHz FSR. (**c**) The central frequency of the comb lines versus the channel number with a linear fitting. Reprinted with permission from Ref. [87].

The driving pulse patterns are designed according to the sweep trace of the tunable filter as shown in Figure 10 [87]. A sequence of super-Gaussian pulses is defined as

(20) $$V_{\mathrm{out}}(t)=V_{A}\times \sum_{i=1}^{N_{p}}\exp\left\{-0.5\times {\left[2\left(t-t_{i}\right)/\tau_{m}\right]}^{8}\right\},$$

where *V*_A_ is the output amplitude, *N*_p_ is the number of pulses, *t_i_* is the peak position of the *i*-th pulse and *τ*_m_ is the duration of an individual pulse. The central temporal position of the *i*-th pulse is given by

(21) $$t_{i}=\arcsin\left[2\left(f_{i}-f_{0}\right)/\Delta f\right]+T/4,$$

where Δ*f* is the spectral sweep range, *f*_0_ is the central frequency, *f_i_* is the frequency of the *i*-th spectral comb line, where *i* is an integer with |*i*| < Δ*f/*2*FSR*, *T* = 1/*f*_s_ is the sweep period and *f*_s_ is the sweep rate of the FFP-TF. Using Equations (20) and (21), reconfigurable pulse patterns with variable comb lines can be designed based on experimental parameters such as the central wavelength, sweep range, sweep period, etc. We have demonstrated k-space uniform comb lines with 50 GHz and 100 GHz FSRs by time-domain modulation and applied them in the OCT imaging system as shown in Figure 12 and Figure 13. It is noted that the quality of discrete swept signals in FDML laser by time-domain modulation is much higher compared with that using comb filters by frequency-domain modulation. These highly stable signals with low timing jitter can be used directly as the clock signal for resampling in OCT systems and potentially in the swept-source time of flight LiDAR. Moreover, time-domain modulation is highly flexible in the generation of reconfigurable discrete comb lines by adjusting the combinations of different pulse trains, which can be applied in the practical OCT imaging systems with long-range detection or fast detection with limited acquisition bandwidth.

**Figure 14:**
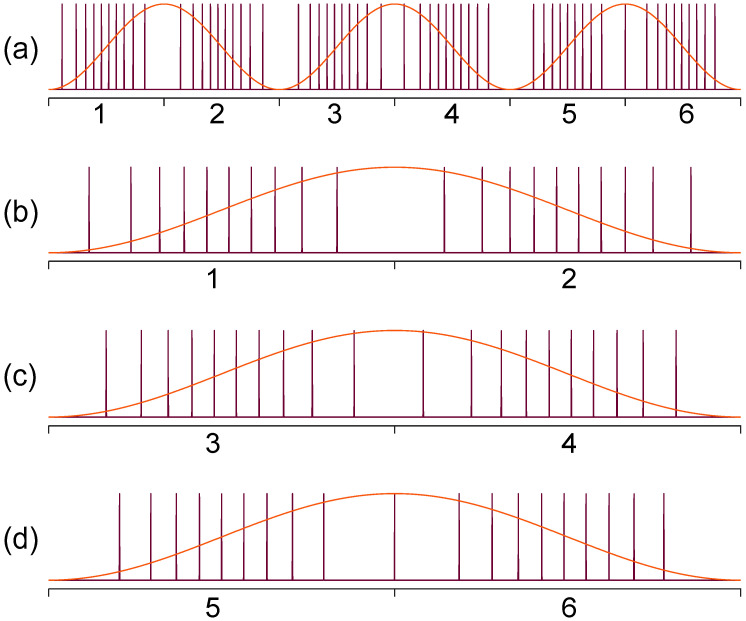
The principle of discrete frequency domain harmonic mode-locked laser with reconfigurable uniform comb lines. (**a**) Six independent pulse train groups with the forward and backward sweeps in three sub-periods, (**b**–**d**) are the three sub-periods with bi-directional sweeps, where the relative shift of the comb lines is shown. Reprinted with permission from Ref. [95].

**Figure 15:**
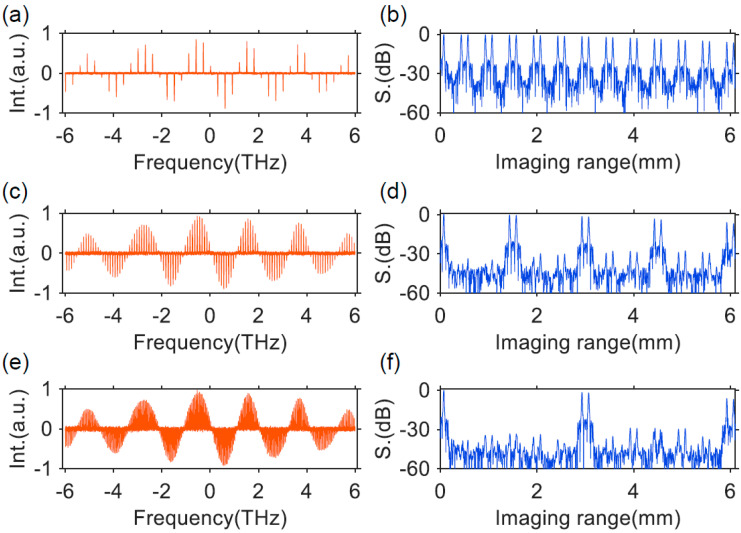
(**a**,**c**,**e**) show the resampled interference fringes in the frequency domain and (**b**,**d**,**f**) show the calculated point spread functions (PSFs) of discrete FDML laser with 300, 100 and 50 GHz FSR. Reprinted with permission from Ref. [95].

The flexibility of temporal modulation is not limited to the manipulation of the pulse distribution and pulse duration of the pulse trains in a sweep. The signal control of this scheme can be extended to using different modulations on the forward and backward sweeps or more than two types of modulations in a single period with a harmonic Fourier domain mode locking [91]. Obviously, such a level of control of the discretization of swept signals is impossible for filter-based FDML lasers. By combining multiple sampling formats, the discrete FDML lasers could provide many advantages to the data processing of OCT. We have proposed and demonstrated a discrete FDML laser with time modulation for simultaneous dual-modal swept-source OCT by driving the tunable filter at the third harmonic frequency of the fiber cavity [95]. The basic principle is that there are three independent sub-periods in the cavity and six groups of sweep when both the forward and backward sweep are considered as shown in Figure 14. Each group sweep (Group 1–6 as shown in Figure 14a) of the pulse trains will generate uniform k-space comb lines with a given FSR, e.g., 300 GHz, in the frequency domain but with stepwise shifted frequency offset with an increment of FSR/6, say 50 GHz, when they are combined in the frequency domain. The discrete comb lines can be used in dual modes where each group sweep (Group 1–6 as shown in Figure 14a) can be individually used with a sweep rate at six times the fundamental frequency while six sweeps can also be grouped together with an FSR/6 comb line and a sweep rate at the fundamental frequency. When applied in an OCT imaging system, a simultaneous dual-mode OCT imaging system in a fast OCT mode and a long-range imaging mode can be achieved by using different processing of the same set of acquired data with a low sampling rate as shown in Figure 15. Such frequency interleaved comb lines are also promising in swept-source LiDAR systems to obtain interleaved line scanning to increase the frame rate.

### 3.6. Summary of FDML Parameters with Different Kinds of Techniques

Compared with conventional tunable lasers, FDML lasers utilize a long fiber delay in the laser cavity to store the whole swept signal and avoid laser rebuilding from the spontaneous emission, which increases the sweep rate to several hundreds of kilohertz or even megahertz if buffering is used. Besides the sweep rate, other parameters of FDML lasers including sweep range, output power, coherence length and stability are also important for FDML based applications such as optical coherence tomography, optical sensing, precision measurement, microwave generation and nonlinear microscopy. The broadband sweep range contains rich spectral information, which will improve the axial resolution in OCT systems, increase measurement precision for optical sensing or broaden the bandwidth in a microwave generation. High output power will increase the penetration depth and signal-to-noise ratio in the imaging for OCT and microscopy systems. Long coherence length will enlarge the detection distance. Laser stability is obviously important for all applications. Different techniques have been proposed to generate high-performance FDML lasers as introduced above, Table 1 summarizes the performance of different techniques to realize FDML lasers.

## 4. Applications of FDML Lasers

FDML lasers have been widely applied in the fastest OCT systems such as field retinal imaging, intravascular imaging, angiographic imaging, eyes imaging, chips imaging, etc. FDML lasers are also promising sources for applications in fiber sensing, precise measurement, microwave generation and nonlinear imaging, which will be presented in this Section.

### 4.1. Optical Coherence Tomography (OCT) Imaging

**Figure 16:**
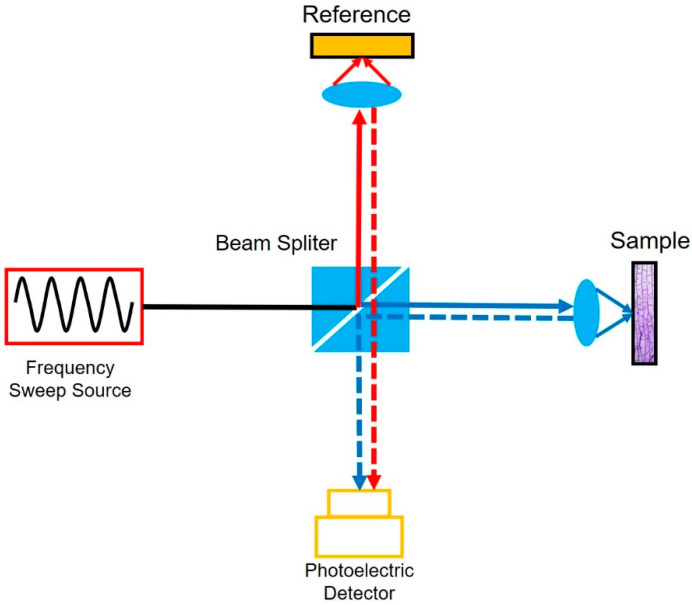
The principle of swept-source OCT.

FDML lasers have been applied in swept-source OCT (SS-OCT) systems with imaging speeds from ~20 kHz to multi-MHz [111,112,113,114,115,116,117,118,119,120]. Figure 16 shows the principle of swept-source OCT. The swept laser is the key component of the SS-OCT system. The swept signal is divided into the reference arm and sample arm. The reflected signals interfere with each other and are detected by a detector. The delay difference between the two arms will introduce periodical modulations in the time domain, which are consistent with the modulations on the spectra as a result of the wavelength to time mapping of the swept source. By resampling the interference signal detected, the interference spectra are obtained and the hierarchical information of the sample is reconstructed by the Fourier transform of the interference spectrum [121]. The performance of the swept sources determines the performance of the SS-OCT systems. The sweep speed, sweep range and the instantaneous linewidth (or coherence length) of the swept source respectively determine the imaging speed, axial resolution and the sensitivity roll-off. FDML lasers with a high sweep rate, wide sweep range and relatively long coherence length are the perfect swept source for SS-OCT systems. At present, FDML lasers have been used for the imaging of human retina and the optic nerve head for ophthalmology [29,94,111,112,113,114,119,122,123,124,125,126], angiographic imaging [93,126,127,128,129], neurosurgery [120] and embryo imaging [116,120,129].

**Figure 17:**
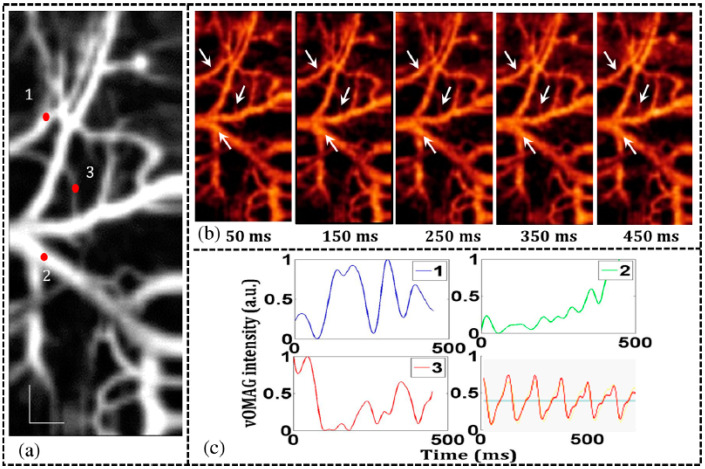
En-face view with sequential numbers indicating (1) an arteriole, (2) a venule, and (3) one of the smallest vessels, respectively. (**b**) Frames taken from the projected 4-D movie (visualization 1) to show time-varying blood flow dynamics. (**c**) OMAG signal variations (spline fitted) to show blood flow dynamics in the functional vessels that marked in (**b**) and sequentially numbered in (**a**). Reprinted with permission from Ref. [128].

High-speed imaging enables many new OCT applications such as angiography, live 3D imaging, large area scans and dynamic flow obstacles [90,93]. An FDML laser with a 1.68 MHz sweep rate is applied in the imaging of retinal angiogram, which can achieve over a 48-degree wide field as shown in Figure 17 [128]. By combining an MHz sweep rate source with a microelectromechanical system (MEMS) scanner, an effective frame of 36 kHz is realized. This high-speed OCT imaging system enables volumetric imaging of a wide-field view of microcirculatory tissue, which provides direct analysis of the flow and details of the sample. This allows for high precision imaging of angiography, which is significant for ocular diagnosis. Capturing the physical movement of the biological samples requires high-speed OCT imaging systems in which MHz FDML lasers can serve as the source. In embryo biology, for example, high-speed imaging with FDML lasers has demonstrated time-resolved imaging of heart motion [68,116,129,130].

Buffered FDML lasers can realize high-speed sweep rates and are more sensitive to the phase noise of the fringe signals in OCT detection systems, which have been applied in phase-sensitive OCT imaging applications. A 3D OCT phase microscopy is demonstrated by a buffered FDML laser, the displacement sensitivities are 39, 52 and 102 pm with 42, 117 and 370 kHz sweep rates, respectively, by subtracting the measured phase of the axial scanning [130]. Megahertz level optical coherence tomography angiography with high contrast has also been proposed and presented by using a coherent buffering FDML laser [93]. The phase is corrected by coherent averaging the buffered signals and therefore the signal-to-noise ratio is enhanced. Besides, a phase linearization algorithm is also applied for the resampling and mitigating of the spectral differences among different buffers. The coherent averaging of different buffers in the FDML laser improves the SNR and sensitivity in optical coherence tomography angiography imaging systems and can also be applied for multiscale imaging with high-speed FDML laser-based OCT systems [93].

### 4.2. Optical Sensing

High-speed spectroscopy has been proven to be a promising tool for real-time measurement based on high speed swept lasers, the wavelengths of which can be converted to temporal positions in the time domain. By detecting the time dependence of the wavelength, we can acquire the absorption or transmission spectrum of the sample to determine properties such as gas pressure, water concentration and gas temperature. A high speed swept laser, like an FDML laser with low noise and relatively narrow instantaneous linewidth, enables real-time hyperspectral absorption spectroscopy for the measurement of multiple gas parameters [131,132]. In 2007, Laura A. et al. demonstrated the crank-angle-resolved gas temperatures based on an FDML laser. The principle is to monitor the high-speed engine gas thermometry through H_2_O absorption spectroscopy. The central wavelength of the FDML laser is chosen at 1350 nm, which coincides with the H_2_O absorption. The laser was also modulated at a 50% duty cycle to allow one of the split pulses as the reference. Utilizing an FDML laser with an acquisition rate of 100 kHz, the gas thermometry can be measured even when the engine rotates at a high speed of 600 to 900 rpm [131]. In addition, by using three FDML lasers and time division-multiplexing, multiple gas parameters including the temperature, pressure, water concentration and gas velocity have been measured [132]. Three FDML lasers at wavelengths 1350.5, 1355.85 and 1365.31 nm together monitor more than two absorption features to average the near-optimum temperature sensitivity. The most temperature-sensitive wavelengths would then be chosen, by virtue of the tunability of the FDML lasers and multiple FDML lasers, which will reveal etalons and interfering absorbers to allow for corrections to multiple parameters. By measuring the H_2_O absorption spectra, the temperature and the concentration are obtained. The gas velocity is acquired by measuring the Doppler shift of the absorption.

**Figure 18:**
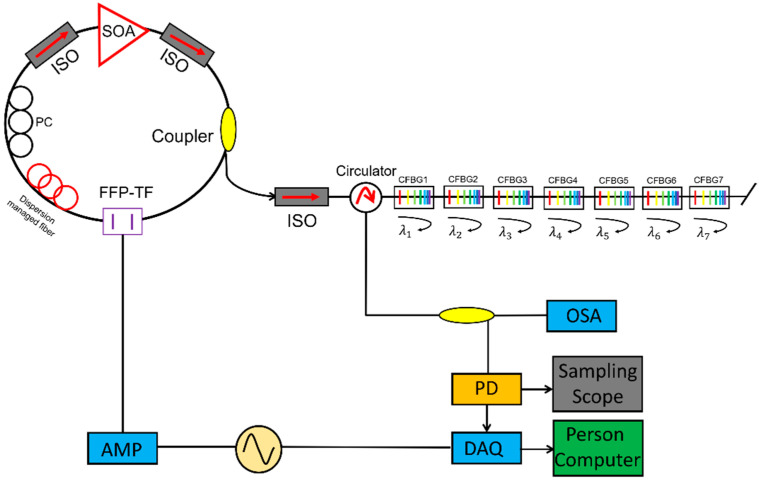
Experimental setup for an FBG sensor array system based on an FDML wavelength-swept laser. Adapted with permission from Ref. [133].

The properties of fiber Bragg gratings (FBGs) such as compactness, resistance to electromagnetic interference and multiplexing capability in a single fiber make FBGs an attractive choice for measuring various physical quantities including strain, temperature and acceleration. High-speed FBG interrogation, high-speed vibrations and real-time dynamic strain monitoring with FDML lasers have been demonstrated [5,6,8,67,102,133,134,135,136,137,138]. Multiple parameters can be measured simultaneously by combining multiple FBGs with different central wavelengths in a single fiber, but multiple lasers with different frequencies are required. The multiple single-frequency lasers can be replaced by a swept laser, which is a powerful tool for high-speed FBG interrogation [133] (Figure 18). High-speed FBG interrogation with a buffered FDML laser [67], linearized interrogation by using an FDML FBG sensor system with a polarization-maintaining fiber Sagnac interferometer [134] and multi-encoding weak FBG interrogation based on an FDML laser [5] have been demonstrated. By using a buffered FDML laser, the sweep rate is 50.7 kHz, enabling a 202.8 kHz measurement rate [67]. The multiple FBGs can measure at a resolution of 4.9 μs without being affected by the delay. Since the FDML laser is driven by a sinusoidal wave, the time to wavelength mapping suffers from the nonlinear filter response in the demodulation. The nonlinear filter response can be corrected by using third-order polynomial fitting for conversion of the filter based on a polarization-maintaining fiber Sagnac interferometer [134]. The high-speed sweep rate of the FDML laser makes it possible to identify the arrival time of the reflections from the multiple FBGs, which can also be applied to the interrogation system of multi-encoding weak FBGs [5].

Different wavelength information can be encoded in the time domain and detected by a high-speed photodetector, thus realizing high-speed sensor multiplexing. Therefore, FDML lasers are also applied to the dynamic FBG strain [139] and high-speed vibration measurement [140]. Jinwoo Park et al. proposed to use an FDML laser combined with six multiplexed FBGs to monitor the dynamic strains [139]. One FBG is used as the reference signal and the others are fixed on the piezoelectric transducer stack to monitor the strain. Real-time measurement is realized by monitoring the variations of the peaks, the intervals of the FBGs and the real-time Fourier transform spectrum. Due to the high speed of the FDML laser, the frequency resolution of the dynamic strain monitoring is 0.5 Hz in 2 s integration time, which has great potential in health monitoring. Similarly, high-speed vibrations at several kilohertz had also been measured by using FBGs with FDML lasers [140].

Besides FBG based sensor systems, FDML lasers have also been applied to the integral hybrid laser temperature sensor and high-speed refractive index sensing inside the laser cavity. C. A. Galindez et al. proposed an integral temperature sensor based on Brillouin laser with the seed from an FDML laser [141]. The driving voltage of the tunable filter in the FDML laser is adjusted to ensure that the maximum optical power is transmitted to the Brillouin ring laser, which is at 1532 nm in the proposed system. A temperature slope of 1.0584 MHz/°C is obtained by measuring the Brillouin shift. Another example of combining laser and sensors is the high-speed refractive index measurement proposed by Yuan Cao et al. [142]. A microfiber Bragg grating is inserted in the laser cavity to acquire the time-domain pulse shift instead of wavelength shift. By using a high-speed photodiode, the high-speed refractive index sensor is realized at 43.06 kHz with 4.6 × 10^−6^ resolution.

Other methods to improve the performance of fiber sensing systems include utilizing Mach-Zehnder interferometer [57], microfiber Bragg grating [142], temperature-controlled Fourier domain mode locking (TC-FDML) [143], polygon-scanner-based wavelength filter [139], recycled residual Raman pump [102], dual-pump fiber optical parametric amplification [103], delayed-peak recognition and calibration [144], delay correction with bidirectional sweep [67], pulse modulation [145] and Fabry–Pérot etalon [146].

### 4.3. Precision Measurement

**Figure 19:**
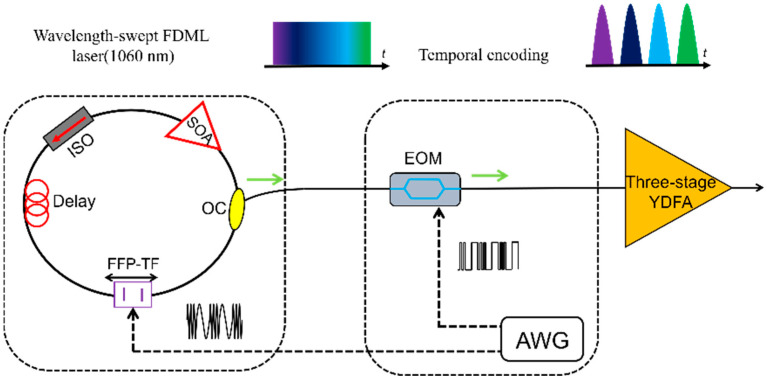
LiDAR based on an FDML laser achieves inertia-free imaging in one dimension with a high number of pixels and flexible imaging parameters. Adapted with permission from Ref. [147].

FDML lasers are widely used in precision measurements, for example, LiDAR, spectrum analyzer and dispersion measurements. Currently, the main bottleneck for the traditional LiDAR system is the mechanical scanning of the source and the detector, which is slow and bulky. In 2020, Yunshan Jiang et al. proposed a time-stretched LiDAR system by using a tunable laser as the scanning source, which makes it possible to use a single laser and a single detector for the measurement. The discrete FDML laser signal with varying central frequencies is directed to different angles to realize the function of a spectrally scanned time-of-flight ranging camera. An FDML laser with a central wavelength at 1060 nm, 10 nm sweep range and 0.342 MHz sweep rate is used in the LiDAR system to realize inertia free LiDAR imaging. The volume of the data generated in three-dimensional imaging is reduced as single detection and high speed swept laser is used as shown in Figure 19 [147].

**Figure 20:**
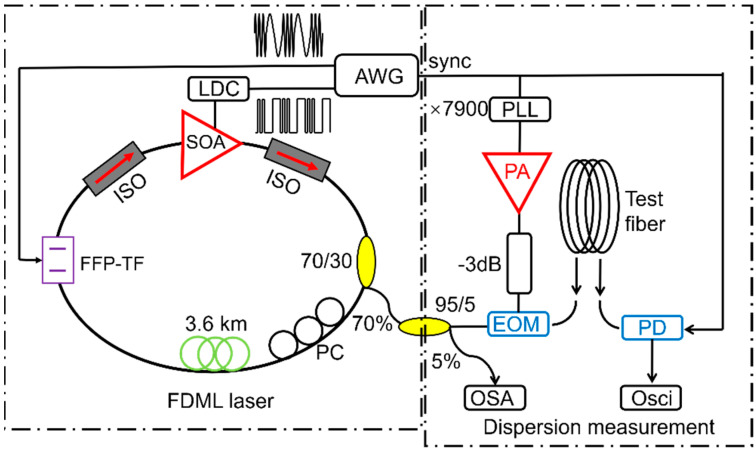
Schematic of the experimental setup to measure the DMGD in a few-mode fiber. Adapted with permission from Ref. [149].

Dispersion or modal group delay is a key parameter of optical fiber, which can be measured by utilizing the wavelength sweeping property of FDML lasers. The dispersion of kilometer level fiber is directly measured by characterizing the propagation delays of different wavelengths of the FDML laser. Higher than 100 Hz frame rates can be realized, thus the measurement is not affected by the thermal shift of the fiber [148]. The differential modal group delay (DMGD) of few-mode fibers can also be measured with FDML lasers, as demonstrated by Varun Kelkar et al. in Figure 20 [149]. Tatsuya Yamaguchi et al. developed a real-time spectroscopy system using an FDML laser. The spectroscopy system performs continuous measurements for 1 min or more with a time resolution of 20 μs [150]. Zhang Xiaomei et al. developed a high-speed spectrum analyzer based on an FDML laser [151]. High-speed spectrum measurement is made possible by a high-speed acquisition system utilizing the wavelength-to-time mapping of the FDML laser, i.e., the spectral information is converted to time information by the high-speed photodiode. This technique can be applied for high-speed and high-precision spectrum acquisition of optical fiber sensor [151].

### 4.4. Microwave Generation

**Figure 21:**
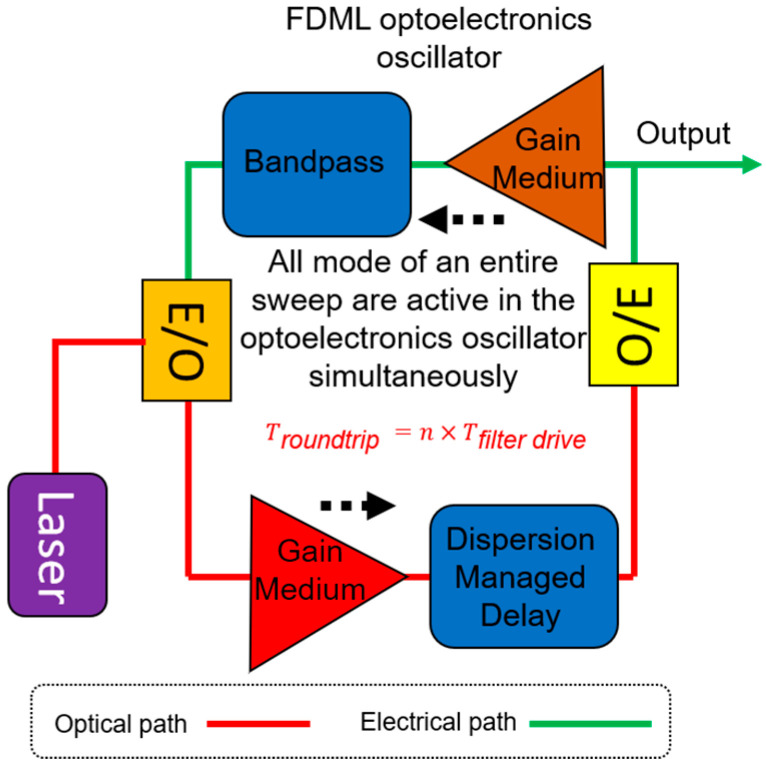
Schematic to show the operations of an OEO based on FDML oscillator. Adapted with permission from Ref. [152].

Traditionally, microwave signals are generated by optoelectronic oscillators (OEOs). A high Q factor element is required in order to reduce phase noise, which in general is difficult to obtain. It is also difficult to generate a chirped microwave waveform using OEO technique. Another method to generate microwave signals is by beating two narrow linewidth laser signals. A chirped microwave signal can be obtained by beating a swept laser signal with a single frequency laser signal. Li et al. demonstrated an FDML OEO scheme to obtain continuously chirped microwave waveforms. All the selected modes are simultaneously stored in the entire sweep similar to the FDML lasers, which can generate chirped microwave waveform because of the sweep as shown in Figure 21 [152]. In addition to the reconfigurable linearly chirped microwave waveform (LCMW), the generation of the dual-chirp microwave waveform [153], LCMW with a large time-bandwidth product [154], phase-coded LCMW [155], frequency-doubled LCMW [156,157], harmonically LCMW [158] and frequency-definable LCMW [159], as well as complementary LCMW pair [160], arbitrary microwave waveform generation [161] and phase-coded microwave signals [162], have also been demonstrated based on the FDML OEO. In addition to the generation of complex microwave waveforms, the FDML OEO can also be used for microwave photonic frequency-to-time mapping [163,164].

Besides using FDML oscillators to generate LCMW, a hybrid FDML laser has also been proposed to generate an ultra-wideband LCMW [154]. A silicon microdisk resonator is used as an optical bandpass filter in the laser cavity to realize frequency domain mode-locking. Therefore, a chirped pulse is generated in an FDML laser, which is further used to generate LCMW by beating with a CW optical carrier from a laser diode. A 50 GHz ultra-wideband LCMW is generated, which can be utilized for high-resolution radar and microwave imaging applications [154].

### 4.5. Nonlinear Microscopy Using FDML Laser

**Figure 22:**
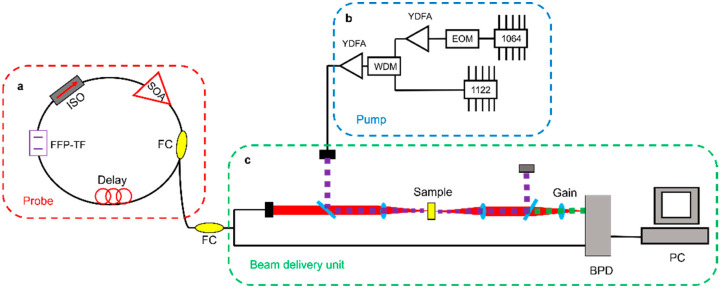
Schematic of the time-encoded Raman system. (**a**) The fibre based, wavelength-swept FDML probe laser25. FC, fibre coupler; FFP-TF, fibre Fabry- Pérot tuneable filter; ISO, optical isolator; SOA, semiconductor optical amplifier. (**b**) The homebuilt fibre-based pump laser is digitally synchronized to the FDML. EOM, electro-optic modulator; WDM, wavelength division multiplexer; YDFA, ytterbium-doped fibre amplifier. (**c**) The lasers are combined in the beam delivery unit and focused onto the sample. The SRG signal is detected after subtraction of the offset by a differential balanced photodetector (BPD). Adapted with permission from Ref. [165].

FDML lasers also can be used in Two-Photon Microscopy and Laser confocal Raman microscope. Sebastian Karpf et al., investigated fingerprint Raman imaging of human tissue sections employing the time-encoded Stimulated Raman scattering microscopy technique by using FDML lasers [165,166,167]. A time encoded Raman concept is proposed as shown in Figure 22, the signal from a pump laser and an FDML probe laser are both focused on the sample [165]. Since the probe laser is a swept laser, the optical energy changes periodically with the swept laser. When the energy difference matches the Raman spectrum of the sample, the intensity of the probe laser is enhanced by stimulated Raman gain, which can be modulated to a Raman spectrum based on time to wavelength encoding of the FDML laser. The encoding concept can be extended to spectro-temporal encoding and applied in multiphoton microscopy with kilohertz frame rates [168]. High-speed scanning can be realized by combining an FDML laser with a diffraction grating. The swept signal from the FDML laser is modulated by a modulator and then directed at the diffraction grating. Each pulse has a distinct pixel in both the time and space domain, which is referred to as spectro-temporal encoding. The proposed technique has realized 342 kHz line scanning rates and 88 MHz pixel rates and a high-quality fluorescence lifetime imaging flow cytometry is demonstrated [168]. Besides Raman amplifiers, a nonlinear imaging scheme using a MOPA-FDML laser [40] was also proposed by Sebastian Karpf et al., A MOPA configuration combined with a second-harmonic generating (SHG) nonlinear crystal is proposed to realize visible wavelength-swept laser with 15 nm sweep range. The visible wavelength-swept laser will open new applications in spectroscopy and high-speed imaging. The proposed SHG method provides a novel scheme to generate wavelength-swept signals in different wavelength regions by making use of the nonlinear process [40].

## 5. Conclusions

FDML laser was firstly proposed in 2005. The sweep rate of FDML lasers can be up to megahertz and the sweep range is more than 100 nm. The relatively short coherence length however is the main drawback. It is known that the residual chromatic dispersion and nonlinearity of the laser cavity induce detuning between the swept signal and the tunable filter, which would trigger instabilities leading to high-frequency fluctuations on the waveforms. To date, it remains challenging to generate stable high quality swept signals in FDML lasers, which is the major difficulty to be overcome in the development of the next generation FDML lasers.

In the theory and modeling of FDML lasers, the major obstacle is the lack of analytical description of the solutions. The only analytical solution is the steady-state solution, which is in general unstable. Numerical simulations could capture the evolutions when conditions are specified. However, observation based on simulations is by nature incomplete and not rigorous. There were attempts to classify the high-frequency fluctuations but an accurate model that can predict the generation of the different high-frequency fluctuation patterns remains lacking. The analytical form and stability study of these high-frequency fluctuation patterns and the engineering and control of them both in modeling and experiments are highly desirable but have not yet been well investigated.

The quest to generate high-speed scanning laser signals with high coherence led to the invention of the long cavity FDML lasers. The relative detuning induced by dispersion is responsible for short coherence length and low stability of swept signal in FDML lasers. Since the invention of the FDML lasers, much of the efforts focus on the elimination of the dispersion by experimental techniques such as dispersion compensation fibers (DCFs), long chirped fiber Bragg gratings (CFBGs) and dynamically controlled CFBGs. However, the high precision required in the dispersion compensation makes it difficult to be implemented and costly for practical applications. Furthermore, DCF is not readily available in wavelengths outside the communication C + L bands. Also, the required ultra-stable temperature control is rather challenging outside the laboratory. Thus, techniques to stabilize the CW solutions of FDML lasers, static, dynamic, or both, that can be used in the field is highly desirable.

The FDML laser is a special kind of laser, with the lasing wavelength varying periodically with time. The spectrum of the swept laser can be obtained by a linear transformation from the temporal waveform captured by a single photodetector. The fundamental dynamics of FDML lasers and their applications in optical fiber sensing, laser metrology, optical coherence tomography (OCT), LiDAR and other applications requiring spectral analysis will continue to be an exciting and fruitful area of research.

## Figures and Tables

**Table 1:** Summary of FDML parameters with different kinds of techniques.

Parameters	Techniques	Output Performance
Sweep rate	High speed tunable filter	419 kHz [90]
	Harmonic mode locking	129.125 kHz [92]
	Buffering	1.67 MHz, 3.35 MHz [111]
Sweep range	Booster SOA	143 nm [42]
	Combination of two SOAs	160 nm [71], 284 nm [73]
Output power	Booster SOA	100 mW [45]
	High gain amplifier	MOPA 2000 mW [105]
	Intra-cavity amplification	12.5 mW [82]
Coherence length	Dispersion compensation (DCF, DSF, CFBG)	The 6-dB roll off length is 5.6 mm for DCF [87], 6.4 mm for DSF [13], and 10.7 mm for CFBG [76]
	Comb filter	The 1.2-dB roll off length is 2.8 mm for F-P comb filter [79], and the 6-dB roll off length is 53 mm for Microring comb filter [80]
Stability	Temperature control	0.001 °C precision [14]
	Cavity length control	0.3125 μm accuracy [85]
	External feedback	Maintain 95% output power [109]
	Time domain modulation	Highly stable pulse [87]

## Data Availability

Not applicable.
